# Impact of Chronic Kidney Disease Severity on COVID-19 Outcomes: A Retrospective Cohort Study

**DOI:** 10.3390/healthcare14162575

**Published:** 2026-08-17

**Authors:** Laura Garazhayeva, Almagul Kauysheva, Maksat Mamyrkul, Mukhit Kulmaganbetov

**Affiliations:** 1 Department of Medicine, Kazakhstan Medical University “KSPH”, Almaty 050000, Kazakhstan; garazhayeva91@mail.ru; 2 National Scientific Centre of Phthisiopulmonology of the Republic of Kazakhstan, Almaty 050000, Kazakhstan; 3 Department of Health Policy and Management, Asfendiyarov Kazakh National Medical University, Almaty 050000, Kazakhstan; 4 Centre for Sanitary and Epidemiological Expertise, Medical Centre of the Presidential Administration of the Republic of Kazakhstan, Astana 010000, Kazakhstan; 5 Department of Molecular Biology and Medical Genetics, Asfendiyarov Kazakh National Medical University, Almaty 050000, Kazakhstan; 6 Centre for Eye and Vision Research, Hong Kong, China

**Keywords:** chronic kidney disease, COVID-19, haemodialysis, in-hospital mortality, acute kidney injury, retrospective cohort study, inflammatory markers, Kazakhstan

## Abstract

**Highlights:**

**What are the main findings?**

- Conservatively managed chronic kidney disease (CKD) patients exhibited the highest in-hospital mortality (34.8%) and acute kidney injury (AKI) rates, significantly surpassing even patients already on maintenance haemodialysis (21.7% mortality).
- Non-dialysis CKD status independently tripled the odds of death (OR 3.23) and quadrupled the odds of AKI (OR 4.12), whereas being on haemodialysis was not an independent predictor of mortality compared to patients without CKD.

**What are the implications of the main findings?**

- Healthcare providers must prioritise aggressive monitoring, early nephrological consultation, and maintain a low threshold for initiating renal replacement therapy in conservatively managed CKD patients hospitalised with severe infections.
- These results provide vital, region-specific evidence for Central Asian and similar transitional healthcare systems to refine triage protocols and resource allocation for distinct renal subgroups during viral respiratory pandemics.

**Abstract:**

Background/Objectives: Chronic kidney disease (CKD) is a recognised risk factor for severe COVID-19, but comparative outcomes between conservatively managed CKD patients and those on maintenance haemodialysis (HD) remain incompletely characterised, particularly in Central Asia. This study evaluated the impact of CKD severity on clinical outcomes, inflammatory profiles, and mortality in hospitalised COVID-19 patients. Methods: This retrospective cohort study reviewed 891 patients hospitalised with COVID-19 at a tertiary infectious disease centre in Almaty, Kazakhstan, between 2020 and 2021. Patients were stratified into three groups: No CKD (*n* = 598), CKD without HD (*n* = 116), and CKD on maintenance HD (*n* = 177). Demographic, laboratory, treatment, and outcome data were extracted from electronic medical records. Multivariate logistic regression was used to identify independent predictors of in-hospital mortality, acute kidney injury (AKI), and intensive care unit (ICU) admission. Model 1 served as the primary prognostic model, while Model 2 explored in-hospital complications. Results: Patients with CKD exhibited significantly higher inflammatory markers, with peak C-reactive protein reaching 168.3 mg/L in the HD group compared to 44.4 mg/L in the No CKD group. In-hospital mortality was highest in the CKD without HD group (34.8%), followed by CKD on HD (21.7%) and No CKD (13.6%). AKI occurred in 35.7% of conservatively managed CKD patients. In multivariate analysis, CKD without HD independently predicted mortality (OR 3.23; 95% CI 1.38–7.63) and AKI (OR 4.12; 95% CI 1.73–10.03). Conversely, established HD status was not a significant independent predictor of mortality after adjusting for age and comorbidities. Conclusions: CKD severity strongly influences COVID-19 prognosis. Paradoxically, conservatively managed CKD patients carry a higher mortality and AKI risk than patients already on maintenance HD, which may be associated with the absence of scheduled volume and metabolic control, although causal inferences cannot be drawn from this observational data. These findings support the need for aggressive monitoring and early nephrological intervention in non-dialysis CKD patients hospitalised with severe viral respiratory infections.

## 1. Introduction

The coronavirus disease 2019 (COVID-19) pandemic, caused by severe acute respiratory syndrome coronavirus 2 (SARS-CoV-2), placed an unprecedented burden on global healthcare systems [1]. While respiratory complications dominate the clinical picture, extrapulmonary involvement, particularly acute kidney injury (AKI), has emerged as a critical determinant of morbidity and mortality [2]. Patients with pre-existing chronic kidney disease (CKD) are especially vulnerable, owing to immune dysregulation, accelerated atherosclerosis, and a high burden of cardiovascular comorbidities [3,4], all of which converge to amplify the risk of poor outcome following viral infection.

In Kazakhstan, the prevalence of CKD and the demand for renal replacement therapy (RRT) have increased significantly over the past decade [5]. The Kazakh nephrology service entered the pandemic with a growing haemodialysis (HD) population and limited reserve capacity in intensive care. During pandemic peaks, nephrology services faced the dual challenge of maintaining chronic dialysis programs while managing AKI in general COVID-19 wards. Several international registries have established that patients with end-stage renal disease (ESRD) on HD have higher mortality rates than the general population when infected with SARS-CoV-2 [6,7]. However, the comparative outcomes of CKD patients managed conservatively (i.e., not yet on dialysis) versus those already on maintenance HD are less well described, and there is a paucity of data from Central Asian healthcare systems.

Conservatively managed CKD patients retain residual renal function, which may be acutely compromised by SARS-CoV-2-induced endothelial injury and cytokine-mediated inflammation, leading to acute-on-chronic kidney injury [8]. In contrast, patients on maintenance HD already have scheduled volume control, regular biochemical monitoring, and established vascular access [9]—factors that may attenuate some of the acute physiological stress of COVID-19 [10]. Understanding how these two populations differ in their inflammatory response, treatment needs, and survival is vital for risk stratification, triage decisions, and resource allocation, particularly in transitional healthcare systems such as Kazakhstan’s.

This study aimed to evaluate the clinical characteristics, laboratory and inflammatory profiles, treatment patterns, and hospital outcomes of COVID-19 patients stratified by renal function status, namely, (1) those without CKD, (2) those with CKD on conservative management, and (3) those on maintenance HD, and to identify independent predictors of in-hospital mortality, AKI, and intensive care unit (ICU) admission. We hypothesised that CKD would be associated with worse COVID-19 outcomes and that the risk profile would differ between conservatively managed and dialysis-dependent subgroups. The findings are intended to inform region-specific clinical guidelines for managing CKD patients during viral respiratory pandemics and to contribute to the limited literature on COVID-19 outcomes in Central Asian nephrology populations.

## 2. Materials and Methods

### 2.1. Study Design and Setting

This retrospective cohort study was conducted at City Clinical Hospital No. 7, a tertiary infectious disease facility in Almaty, Kazakhstan, designated as a regional COVID-19 treatment centre during the pandemic. The study included patients hospitalised with confirmed (positive reverse-transcription polymerase chain reaction for SARS-CoV-2) or clinically diagnosed COVID-19 (typical computed tomography findings and compatible epidemiological history) between 2020 and 2021. Data were extracted from the hospital’s electronic medical records (EMRs) and retrospectively analysed. The study was conducted in accordance with the Declaration of Helsinki, and the requirement for individual informed consent was waived owing to the retrospective, anonymised nature of the analysis. All patient records were de-identified prior to analysis.

### 2.2. Sample and Sampling Strategy

The study population initially comprised 898 consecutive patients admitted to the infectious disease and provisional departments of the hospital during the study period. After excluding 7 patients with incomplete data on key outcomes (mortality or renal status) prior to stratification, the final analysed cohort of 891 patients was stratified into three groups based on renal status: Group 1 (No CKD, *n* = 598), Group 2 (CKD without HD/conservative management, *n* = 116), and Group 3 (CKD on maintenance HD, *n* = 177). CKD status was determined based on documented medical history and estimated glomerular filtration rate (eGFR) values [11], calculated using the Chronic Kidney Disease Epidemiology Collaboration (CKD-EPI) equation [12], with CKD defined as eGFR < 60 mL/min/1.73 m^2^ or an established diagnosis of structural kidney disease for at least three months. Patients on maintenance HD were those receiving thrice-weekly HD for ESRD prior to the index admission. Patients with incomplete data on key outcomes (mortality or renal status, *n* = 7) were excluded from the primary analysis for those specific variables only; all available data were retained for descriptive analyses.

### 2.3. Data Collection

Data extraction was performed by two trained investigators using a standardised electronic case report form. Variables included demographic information (age, sex), comorbidities (diabetes mellitus, arterial hypertension, chronic heart failure, obesity, chronic obstructive pulmonary disease, prior stroke, prior myocardial infarction), admission vital signs and disease severity (peripheral oxygen saturation [SpO_2_], respiratory rate, COVID-19 severity category), in-hospital treatments (oxygen therapy, non-invasive ventilation [NIV], invasive mechanical ventilation [IMV], vasopressor support, antiviral and/or antibacterial therapy, anticoagulation with heparin or enoxaparin, glucocorticoids), and clinical outcomes (ICU admission, AKI, acute respiratory distress syndrome [ARDS], multi-organ dysfunction syndrome [MODS], in-hospital death, hospital length of stay). COVID-19 severity was classified according to the Kazakhstan national COVID-19 management protocol into mild, moderate, severe, and extremely severe categories [13]. AKI was diagnosed according to the Kidney Disease: Improving Global Outcomes (KDIGO) criteria [9,14].

### 2.4. Laboratory and Inflammatory Markers

Laboratory values were analysed as admission values and peak values recorded during hospitalisation to capture the maximum severity of the inflammatory response and organ dysfunction. The panel included haemoglobin, platelets, serum creatinine, urea, albumin, alanine aminotransferase (ALT), aspartate aminotransferase (AST), lactate dehydrogenase (LDH), C-reactive protein (CRP), D-dimer, fibrinogen, ferritin, and procalcitonin. For serum creatinine, three time points were recorded: admission value, peak value during hospitalisation, and last value before discharge or death. Worsening renal function was defined as an increase in peak creatinine above the admission value, and recovery was defined as a decrease in creatinine from the peak value at any subsequent measurement.

### 2.5. Statistical Analysis

Statistical analysis was performed using the R programming language (version 4.3.2; R Foundation for Statistical Computing, Vienna, Austria) using the “stats”, “car” (for VIF), and “pROC” (for AUC) packages. Variables were selected for inclusion based on clinical relevance and the established literature rather than automated stepwise selection, with events-per-variable ratios considered to prevent overfitting. Prior to analysis, the database was screened for the number of observations, variable types, value ranges, duplicates, logical consistency, and missing data. Extreme laboratory outliers were assessed visually and retained as they reflect true pathophysiological severity in this critically ill cohort rather than data errors. Categorical variables were standardised to account for heterogeneous coding of binary responses (e.g., “yes/no”, “1/0”, or text variants) in the EMRs. Continuous variables were assessed for normality both visually and analytically; given the expected non-normal distribution of most laboratory values, continuous variables are presented as medians with interquartile range (IQR) and categorical variables as frequencies *n*/*N* (%).

Intergroup differences across CKD status groups were assessed using the Kruskal–Wallis test for continuous variables and the Pearson *χ*^2^ test or Fisher’s exact test for categorical variables, depending on expected cell frequencies. Multivariate logistic regression was used to identify factors associated with three primary outcomes: in-hospital mortality, AKI, and ICU admission/treatment. Results are reported as odds ratios (ORs) with 95% confidence intervals (CIs). For mortality, a two-stage modelling approach was used: Model 1 included baseline demographic and clinical factors available at admission (age, sex, CKD status, diabetes, hypertension, heart failure, obesity, SpO_2_ on admission, day of illness at admission) to serve as the primary prognostic tool. Model 2 additionally incorporated in-hospital clinical severity markers and complications (AKI, peak creatinine, peak CRP, peak D-dimer, IMV, ARDS, MODS) as an exploratory explanatory model to evaluate the incremental contribution of dynamic in-hospital events rather than for baseline prognostication.

Model performance was assessed using the Akaike Information Criterion (AIC), McFadden’s pseudo-*R*^2^, and the area under the receiver operating characteristic curve (AUC-ROC). Discrimination was categorised as acceptable (AUC 0.7–0.8), excellent (0.8–0.9), or outstanding (≥0.9). Potential multicollinearity was evaluated using the maximum variance inflation factor (VIF); clinically overlapping variables were not entered into the same model when VIF exceeded 5. Missing data were evaluated for all key analytical variables; variables with substantial missingness (e.g., SpO_2_ missing in >50% of records, peak D-dimer in 19.5%) were excluded from the multivariable models. The primary analysis followed a complete-case approach for regression models to ensure stable estimates, while descriptive tables used available-case analysis. All statistical tests were two-sided, and *p*-values < 0.05 were considered statistically significant.

Given the exploratory and descriptive nature of the baseline intergroup comparisons across Table 1, Table 2, Table 3 and Table 4, *p*-values were not adjusted for multiple testing (e.g., Bonferroni correction) and should be interpreted as indicators of group differences rather than strictly confirmatory hypotheses.

### 2.6. Ethical Considerations

The study was conducted in accordance with the Declaration of Helsinki. As a retrospective analysis of fully anonymised data, the requirement for individual informed consent was waived by the Institutional Review Board of Kazakhstan Medical University “KSPH” (protocol code IRB-A322 on 8 November 2023). All patient data were de-identified prior to analysis to protect privacy.

## 3. Results

### 3.1. Baseline Demographic and Clinical Characteristics

The study cohort comprised 891 hospitalised COVID-19 patients, of whom 598 (67.1%) had no CKD, 116 (13%) had CKD on conservative management, and 177 (19.9%) had CKD on maintenance HD. The demographic and clinical characteristics of the three groups are summarised in Table 1. The median age of the total cohort was 61.0 years (IQR 50.0–71.0). Significant demographic differences were observed across groups: the HD group was younger (median 59.0 years, IQR 48.0–66.0) than the conservative-CKD group (median 66.0 years, IQR 56.8–73.0; *p* < 0.001), reflecting the demographic profile of the Kazakhstan ESRD population. The HD group also had a higher proportion of males (61%) compared with the No-CKD (44.8%) and CKD-without-HD (48.3%) groups (*p* = 0.001).

Notably, the median SpO_2_ on admission was 90% across all groups, which is likely a documentation artefact from pandemic-era triage, where hypoxic values were often capped at 90% in electronic records; the statistically significant difference is driven by the differing interquartile ranges (e.g., 86–90% vs. 90–95%). Comorbidities were significantly more prevalent in the CKD groups than in the No-CKD group. Diabetes mellitus was present in 60.9% of CKD-without-HD patients, 36.2% of HD patients, and 27.6% of No-CKD patients (*p* < 0.001). Arterial hypertension followed a similar gradient, affecting 86.2% of conservative-CKD, 92.1% of HD, and 50.3% of No-CKD patients (*p* < 0.001). Chronic heart failure was diagnosed in 67.2% of CKD-without-HD, 55.9% of HD, and 33.6% of No-CKD patients (*p* < 0.001). Prior stroke and prior myocardial infarction were significantly more common in the CKD-without-HD group (23.3% and 8.7%, respectively). Disease severity at admission was high across the cohort: 82.2% of all patients presented with severe COVID-19 and 3.6% with extremely severe disease, with the highest proportion of extremely severe cases observed in the CKD-without-HD group (11.5%). Hospital length of stay was significantly longer in the HD group (median 10 days, IQR 7.0–13.0) than in the No-CKD group (median 7 days, IQR 5.0–10.0; *p* < 0.001).

### 3.2. Treatment and Respiratory Support

Treatment intensity paralleled disease severity across CKD groups (Table 2). Supplemental oxygen therapy was administered to 82.2% of the overall cohort, with the highest rate observed in the HD group (93.2%), followed by the CKD-without-HD group (87.9%) and the No-CKD group (77.9%) (*p* < 0.001). Non-invasive ventilation (NIV) was required in 8.2% of patients overall, with significantly higher utilisation in both CKD groups (14.2% in CKD-without-HD and 17.5% in HD vs. 4.4% in No-CKD; *p* < 0.001). Invasive mechanical ventilation (IMV) was used in 16.5% of patients overall, with the highest rate in the CKD-without-HD group (28.4%) and an intermediate rate in the HD group (22.7%) compared with No-CKD (12.4%; *p* < 0.001).

ICU admission or transfer occurred in 26.7% of patients overall and was significantly more frequent in the CKD-without-HD group (41.3%) than in the HD (32.7%) and No-CKD (21.1%) groups (*p* < 0.001). Vasopressor/adrenergic support was required in 38.8% of CKD-without-HD patients, 24.9% of HD patients, and 16.6% of No-CKD patients (*p* < 0.001). Antiviral therapy (favipiravir or remdesivir) was used infrequently (1.7%) and did not differ across groups. Antibacterial therapy was administered to 96.4% of patients overall and to all HD patients (100%). Anticoagulation with enoxaparin was significantly more common in both CKD groups (62.9% and 66.7%) than in the No-CKD group (45.5%; *p* < 0.001), whereas unfractionated heparin use did not differ significantly. Glucocorticoids were administered to 82.4% of patients overall, with no significant difference across groups (*p* = 0.281).

### 3.3. Laboratory and Inflammatory Profiles

Patients with CKD exhibited significantly elevated inflammatory and renal markers compared with those without CKD, with the most pronounced abnormalities observed in the HD group (Table 3). The median peak CRP was markedly higher in the HD group (168.3 mg/L, IQR 28.9–327.4) and the CKD-without-HD group (110.4 mg/L, IQR 10.9–271.2) than in the No-CKD group (44.4 mg/L, IQR 10.0–178.5) (*p* < 0.001). Admission CRP showed a similar gradient, reaching 76.8 mg/L in the HD group versus 31.7 mg/L in the No-CKD group (*p* < 0.001). Peak ferritin levels were nearly threefold higher in the HD group (1109.0 µg/L, IQR 603.5–2158.4) than in the No-CKD group (383.8 µg/L, IQR 181.5–772.3; *p* < 0.001), and peak procalcitonin was approximately fifteen-fold higher in HD patients (1.5 ng/mL, IQR 0.6–2.9) than in No-CKD patients (0.1 ng/mL, IQR 0.1–0.3; *p* < 0.001).

**Figure 1:**
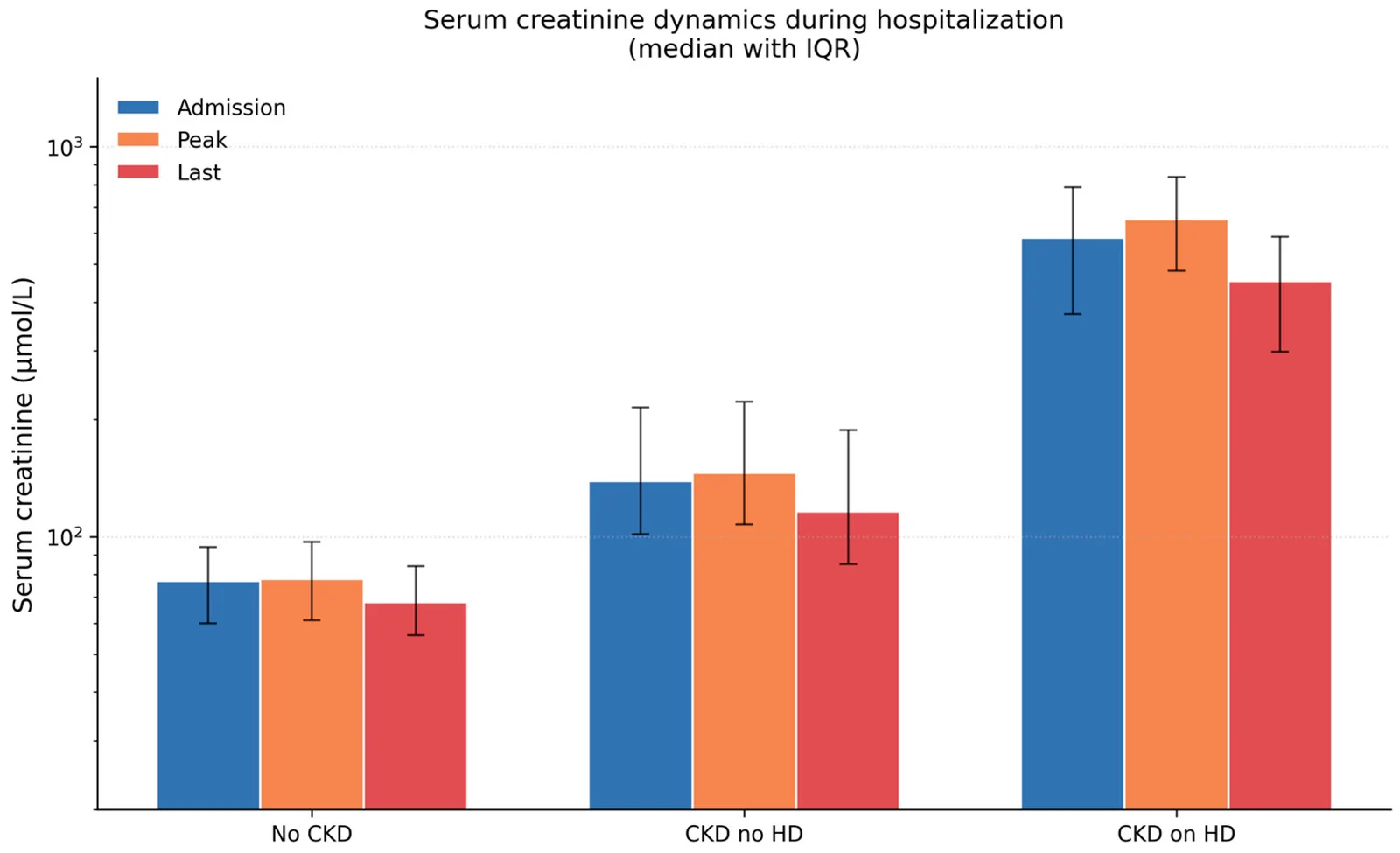
Serum creatinine dynamics during hospitalisation by CKD status. Bars show median values; error bars indicate the interquartile range (Q1–Q3). Note the log-scale y-axis. HD: haemodialysis.

**Figure 2:**
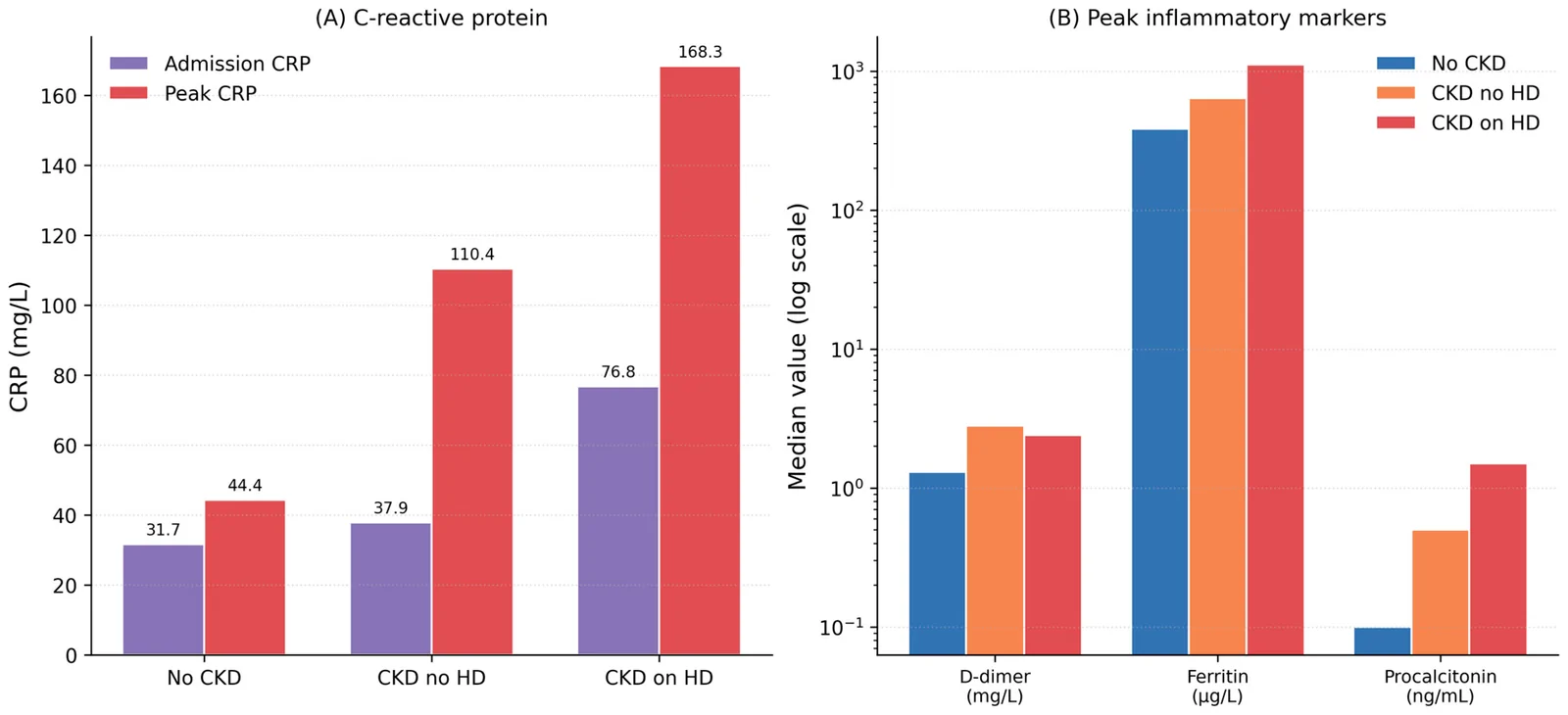
Inflammatory marker profiles by CKD status. (**A**) C-reactive protein (CRP) at admission and peak. (**B**) Peak D-dimer, ferritin, and procalcitonin (log scale). HD: haemodialysis.

Peak D-dimer was significantly elevated in both CKD-without-HD (2.8 mg/L, IQR 1.2–7.1) and HD (2.4 mg/L, IQR 1.2–5.9) compared with No-CKD (1.3 mg/L, IQR 0.6–3.7; *p* < 0.001), indicating a heightened prothrombotic state. Serum creatinine at admission differed sharply across groups: 77.0 µmol/L (IQR 60.0–94.0) in No-CKD, 139.0 µmol/L (IQR 101.5–214.5) in CKD-without-HD, and 584.0 µmol/L (IQR 372.0–788.2) in HD patients (*p* < 0.001), with peak creatinine reaching 652.5 µmol/L in the HD group. Urea at admission and peak urea showed the same gradient. Haemoglobin was lowest in the HD group (105.0 g/L, IQR 91.5–119.0), consistent with the chronic anaemia of ESRD. Peak LDH was significantly elevated in both CKD groups, reflecting greater tissue injury. The dynamic profiles of serum creatinine and inflammatory markers during hospitalisation are illustrated in Figure 1 and Figure 2, respectively.

### 3.4. Clinical Outcomes

**Figure 3:**
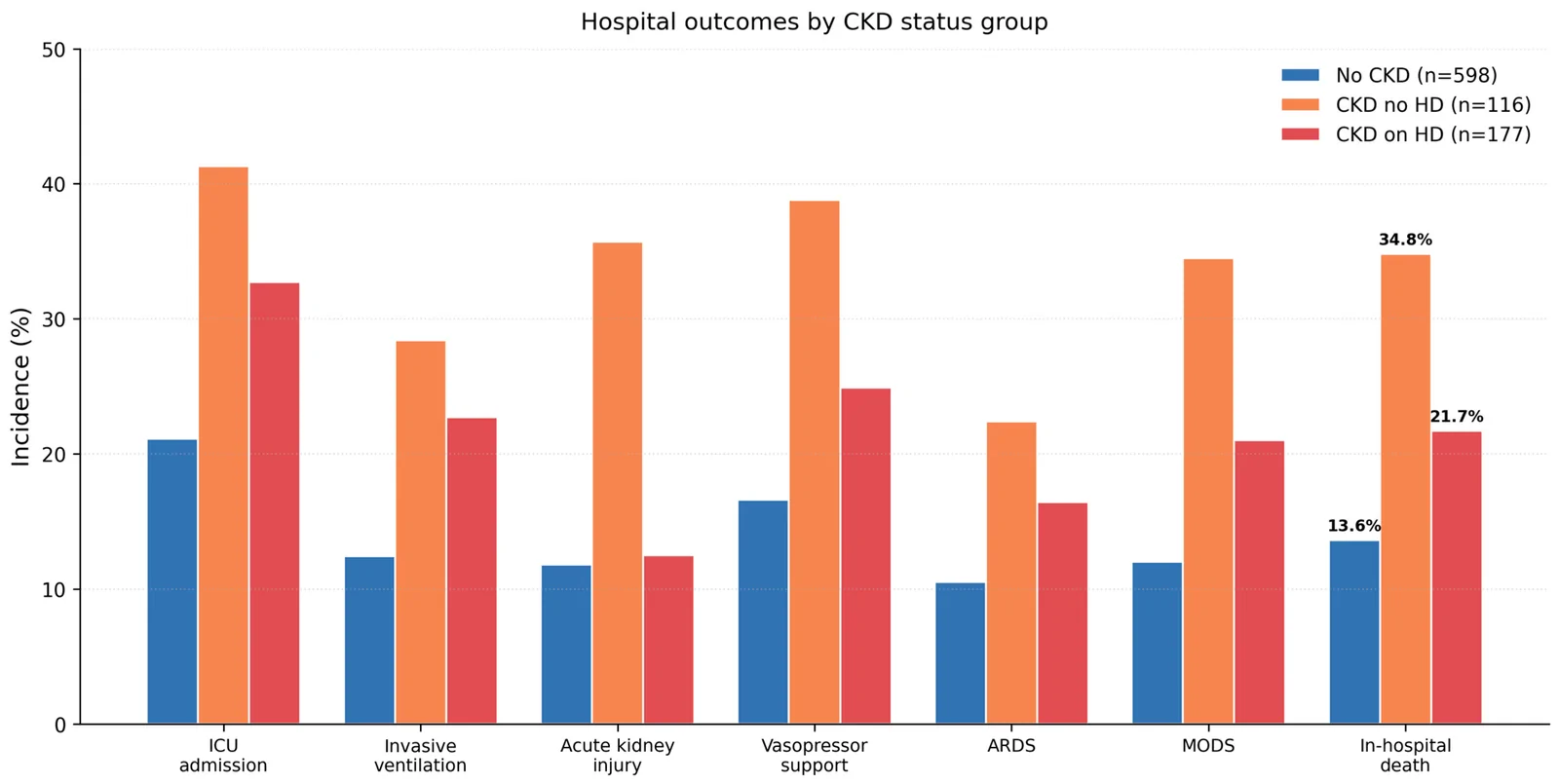
Hospital outcomes by CKD status group. Incidence of intensive care unit (ICU) admission, invasive mechanical ventilation (IMV), acute kidney injury (AKI), vasopressor support, acute respiratory distress syndrome (ARDS), multi-organ dysfunction syndrome (MODS), and in-hospital death across the three renal status groups.

Clinical outcomes are summarised in Table 4 and Figure 3. Severe or extremely severe COVID-19 at any point during hospitalisation was documented in 85.9% of the overall cohort, with the highest rates in the CKD groups (92.5% in HD and 91.2% in CKD-without-HD vs. 82.9% in No-CKD; *p* = 0.001). AKI developed in 132 of 891 patients (14.8%) overall and was strikingly more frequent in the CKD-without-HD group (35.7%) than in the HD (12.5%) and No-CKD (11.8%) groups (*p* < 0.001). The comparatively low AKI rate in HD patients likely reflects the established anuria or scheduled dialysis that precludes the clinical recognition of new AKI by creatinine-based criteria. Worsening renal function (defined as peak creatinine exceeding admission creatinine) occurred in 10.8% of HD patients, 6.1% of CKD-without-HD patients, and 2.1% of No-CKD patients (*p* < 0.001), whereas subsequent creatinine recovery was observed in 75% of HD patients, 58.3% of CKD-without-HD patients, and 40.8% of No-CKD patients.

ARDS developed in 13.2% of the cohort overall and was significantly more frequent in the CKD-without-HD group (22.4%) than in the HD group (16.4%) and the No-CKD group (10.5%) (*p* = 0.001). MODS followed a similar pattern (34.5% in CKD-without-HD, 21% in HD, 12% in No-CKD; *p* < 0.001). The most striking finding was the mortality gradient: in-hospital mortality occurred in 17.8% of the overall cohort, with the highest rate in the CKD-without-HD group (34.8%), followed by the HD group (21.7%) and the No-CKD group (13.6%) (*p* < 0.001). Hospital length of stay was significantly longer in the HD group (median 10 days) than in the No-CKD group (median 7 days; *p* < 0.001).

### 3.5. Predictors of In-Hospital Mortality

Multivariate logistic regression identified CKD status as a key independent predictor of in-hospital mortality, but with an unexpected pattern across CKD subgroups (Table 5). In Model 1 (baseline clinical factors available at admission, *n* = 329), age was independently associated with mortality (OR 1.03 per year; 95% CI 1.01–1.06; *p* = 0.016), as was CKD-without-HD status (OR 3.23; 95% CI 1.38–7.63; *p* = 0.007). In contrast, CKD on HD was not a significant predictor (OR 1.03; 95% CI 0.44–2.31; *p* = 0.951). Diabetes, hypertension, heart failure, obesity, SpO_2_ at admission, and day of illness at admission were not independent predictors in this model.

In the extended Model 2, which additionally incorporated in-hospital complications and dynamic severity markers, the model achieved outstanding discrimination (AUC = 0.999; McFadden’s pseudo-*R*^2^ = 0.393) but with very wide confidence intervals for several predictors (e.g., ARDS, MODS, IMV), reflecting quasi-complete separation driven by the near-perfect fatality of multi-organ failure in this cohort. Furthermore, Model 2 exhibited severe multicollinearity (maximum VIF = 10.79); therefore, the individual coefficients in Model 2 should not be interpreted quantitatively. The model is presented solely to illustrate the near-deterministic relationship between late-stage complications and death; it is strictly exploratory and should not be interpreted or used as a validated predictive model. Model 1 was therefore selected as the primary prognostic model for clinical interpretation.

### 3.6. Predictors of AKI and ICU Admission

CKD-without-HD status was the strongest independent predictor of AKI (OR 4.12; 95% CI 1.73–10.03; *p* = 0.002), whereas CKD on HD was not (OR 0.71; 95% CI 0.25–1.87; *p* = 0.493) (Table 6). Chronic heart failure showed a trend toward higher odds of AKI (OR 2.43; 95% CI 0.94–6.74; *p* = 0.074). Peak D-dimer was borderline associated with AKI (OR 1.02 per 0.1 mg/L; 95% CI 1.00–1.05; *p* = 0.067). Age, sex, diabetes, hypertension, SpO_2_ at admission, peak CRP, and glucocorticoid use were not independently associated with AKI.

For ICU admission (Table 7), independent predictors were age (OR 1.03 per year; 95% CI 1.00–1.06; *p* = 0.045), in-hospital AKI (OR 4.55; 95% CI 1.95–11.04; *p* < 0.001), and peak D-dimer (OR 1.04; 95% CI 1.01–1.09; *p* = 0.034). Notably, neither CKD-without-HD (OR 1.79; 95% CI 0.63–5.07; *p* = 0.273) nor CKD on HD (OR 0.61; 95% CI 0.14–2.44; *p* = 0.502) were independent predictors of ICU admission after adjustment, suggesting that the higher observed ICU utilisation in CKD patients is mediated through intermediate events such as AKI and hypercoagulability rather than by CKD status itself.

### 3.7. Model Diagnostics

Model performance is summarised in Table 8. The baseline mortality model (Model 1) demonstrated acceptable discrimination (AUC = 0.755) and fit (AIC = 303.40; McFadden’s pseudo-*R*^2^ = 0.160) with low multicollinearity (all individual variable VIFs < 1.5; maximum VIF = 1.34). While formal calibration plots (e.g., Hosmer–Lemeshow) were not conducted due to the retrospective nature and complete-case constraints, Model 1 was selected as the primary prognostic model due to its stability and lack of separation. The extended mortality model (Model 2) achieved near-perfect discrimination (AUC = 0.999) and very high pseudo-*R*^2^ (0.393), but at the cost of substantial multicollinearity (maximum VIF = 10.79) and quasi-complete separation for ARDS, MODS, and IMV, a known limitation when outcome events are nearly deterministic for these predictors. The AKI model showed acceptable discrimination (AUC = 0.759; pseudo-*R*^2^ = 0.155), and the ICU model showed excellent discrimination (AUC = 0.823; pseudo-*R*^2^ = 0.195).

## 4. Discussion

This study provides a detailed analysis of COVID-19 outcomes stratified by CKD severity in a large cohort treated at a major tertiary centre in Almaty, Kazakhstan. The findings corroborate international evidence that CKD is a major risk factor for severe COVID-19 [6,7,8]. However, the differential risk within CKD subgroups offers novel insights for clinical management in Central Asian populations. The most striking observation was the higher mortality rate in the CKD-without-HD group (34.8%) compared with the CKD-on-HD group (21.7%), despite the latter having higher baseline inflammatory markers (peak CRP 168.3 vs. 110.4 mg/L) and more advanced kidney disease. In the adjusted Model 1, CKD-without-HD independently tripled the odds of in-hospital death (OR 3.23; 95% CI 1.38–7.63), whereas HD status did not significantly differ from the No-CKD reference (OR 1.03; 95% CI 0.44–2.31).

This “mortality paradox” aligns with findings from international registries [15,16,17,18], confirming that conservatively managed CKD patients are exceptionally vulnerable. However, our study adds vital region-specific evidence from a Central Asian transitional healthcare system, where baseline demographic profiles (e.g., a younger HD population) and resource limitations during pandemic surges present unique clinical challenges. While causal mechanisms cannot be established from this retrospective observational design, one hypothesis for this observation involves acute-on-chronic kidney injury, in which the systemic stress of SARS-CoV-2 infection (cytokine release, endothelial dysfunction, microthrombosis, and hemodynamic instability) precipitates rapid decompensation in vulnerable kidneys that still possess residual function [8]. Patients on maintenance HD may benefit from strict volume control, scheduled metabolic clearance, regular biochemical monitoring, and established vascular access [9,19], factors which may be associated with attenuated acute cardiopulmonary stress of COVID-19 [20,21], though a direct protective causal effect cannot be confirmed. Conversely, conservatively managed CKD patients do not routinely receive this external homeostatic support, which may contribute to an elevated risk of fluid overload [22], electrolyte imbalances [23], uremic complications [24], or unrecognised AKI [25] superimposed on chronic dysfunction. The 35.7% AKI rate in CKD-without-HD patients, nearly 3 times that of the No-CKD group, is consistent with this hypothesis.

The markedly elevated levels of CRP, D-dimer, ferritin, and procalcitonin observed in CKD patients, particularly those on HD, confirm a hyperinflammatory phenotype consistent with the “cytokine storm” of severe COVID-19 [26,27]. The peak CRP of 168.3 mg/L in HD patients is among the highest reported in the literature and likely reflects both the acute SARS-CoV-2 response and the chronic inflammatory milieu of ESRD. The strong association between peak D-dimer and both ICU admission (OR 1.04 per 0.1 mg/L) and borderline AKI (OR 1.02) reinforces the central role of coagulation activation in COVID-19-related organ injury. For healthcare providers in Kazakhstan and similar settings, these findings underscore the value of routine monitoring of inflammatory and coagulation markers in CKD patients with COVID-19, both for prognostic stratification and for tailoring anticoagulation intensity. The significantly higher use of enoxaparin in both CKD groups (62.9% and 66.7% vs. 45.5% in No-CKD) suggests that clinicians already recognised this elevated thrombotic risk during the study period.

Treatment intensity paralleled disease severity across groups. The universal use of antibacterial therapy in the HD group (100%) [28] and very high use in the CKD-without-HD group (96.6%) likely reflect both documented or suspected bacterial superinfection and the prophylactic practice common in severe COVID-19 management [29] during the early pandemic. Glucocorticoid use was high and uniform across groups (≈82–86%), consistent with the adoption of RECOVERY-style protocols [30]. The higher IMV rate in CKD-without-HD (28.4%) compared with HD (22.7%) is notable and may reflect either delayed referral, more aggressive disease progression, or a lower threshold for intubation in conservatively managed CKD patients who lack scheduled dialysis support. The longer hospital stay in HD patients (median 10 days) reflects the complexity of managing dialysis in inpatient COVID-19 cohorts, including isolation logistics and dialysis staff scheduling.

The independent association between CKD-without-HD and AKI (OR 4.12; 95% CI 1.73–10.03) is biologically plausible and clinically important. These patients retain residual renal function that can be acutely compromised, whereas HD patients, by definition, are anuric or oliguric and cannot develop a creatinine-based AKI diagnosis in the same way. Furthermore, standard creatinine-based KDIGO criteria for AKI have inherent limitations in patients already on maintenance HD, precluding the clinical recognition of new-onset AKI in these patients and explaining their artificially low AKI incidence compared to conservatively managed CKD patients. The borderline association of chronic heart failure with AKI (OR 2.43; *p* = 0.074) is consistent with the cardiorenal syndrome literature [31,32]. For ICU admission, AKI itself was the strongest predictor (OR 4.55; 95% CI 1.95–11.04), followed by peak D-dimer and age, whereas CKD status was not an independent predictor after adjustment. This suggests that the higher observed ICU utilisation in CKD patients is mediated through intermediate clinical events (AKI, hypercoagulability) rather than by CKD status itself, which has implications for triage: CKD patients without these intermediate events may not require empiric ICU admission but should be monitored closely for their development.

This study has several strengths. It is one of the largest single-centre CKD-COVID-19 cohorts reported from Central Asia (*n* = 891), with detailed stratification into three renal status groups and a comprehensive panel of admission and peak laboratory values. The use of multivariate regression with formal model diagnostics (AUC, AIC, VIF) provides a transparent assessment of model quality and identifies the limitations of the extended mortality model. The two-stage modelling approach allows separate evaluation of baseline versus dynamic predictors, which is methodologically useful for early prognostication.

Several limitations should be acknowledged. First, the retrospective, single-centre design may restrict generalizability to other Kazakhstan regions or to rural settings with different patient mixes and resource constraints. Second, although we adjusted for major comorbidities, the CKD-without-HD group was fundamentally older and sicker at baseline (higher rates of obesity, diabetes, CHF, and stroke). Despite multivariable adjustment, residual confounding from this heavy baseline comorbidity burden, as well as unmeasured factors like medication adherence prior to admission, specific aetiologies of CKD (diabetic nephropathy, hypertensive nephrosclerosis, glomerular disease), dialysis vintage, and pre-admission functional status cannot be excluded. Propensity score matching was considered but deemed inappropriate due to the distinct physiological baseline differences between CKD subtypes and the further reduction in sample size it would require. Furthermore, a critical limitation when comparing conservatively managed CKD patients with those on maintenance HD is the potential influence of survivor bias. Patients established on maintenance HD have, by definition, survived long enough to reach end-stage renal disease and initiate dialysis, whereas the conservatively managed CKD cohort may inherently include frailer patients who would not survive to reach the dialysis stage. This inherent survivor bias in the HD population, combined with residual confounding from unmeasured differences in baseline frailty, specific CKD aetiologies, and pre-admission functional status, limits our ability to draw causal conclusions regarding the comparative survival of these groups. Third, some laboratory variables had substantial missingness (e.g., SpO_2_ missing in 60.7% of records, peak D-dimer in 19.5%), reflecting the operational realities of pandemic-era charting [33]. We addressed this by complete-case analysis for regression models and by excluding high-missingness variables from the multivariable models, which may limit the generalizability of the adjusted estimates. Fourth, the extended mortality model (Model 2) suffered from quasi-complete separation [34] due to the very high case fatality of multi-organ failure and ARDS, as well as severe multicollinearity (VIF > 10); therefore, these results should be interpreted qualitatively rather than quantitatively [34]. Fifth, *p*-values for baseline intergroup comparisons across Table 1, Table 2, Table 3 and Table 4 were not adjusted for multiple testing, meaning some statistically significant differences may represent Type I errors. Finally, formal calibration assessments (e.g., deciles of risk, Hosmer–Lemeshow tests) were not conducted due to the retrospective design and reduced sample sizes in complete-case multivariable models, which limits the assessment of model linearity and calibration. Furthermore, the study covers 2020–2021 and does not capture later variants of concern or the impact of vaccination, which became widespread in Kazakhstan from mid-2021 onward [35].

## 5. Conclusions

CKD severity strongly influences COVID-19 prognosis. The principal finding of this retrospective cohort study is that patients with CKD managed conservatively (not yet on maintenance haemodialysis) are at exceptionally high risk of in-hospital mortality (34.8%) and acute kidney injury (35.7%), independently of age and major comorbidities, and that this risk exceeds that of patients already on maintenance haemodialysis (21.7% mortality). Although their baseline unadjusted mortality was higher than non-CKD patients, dialysis status did not independently predict death after multivariable adjustment. The observed lower adjusted mortality in HD patients compared to conservatively managed CKD patients may be associated with scheduled volume control, regular biochemical monitoring, and established vascular access, whereas conservatively managed CKD patients are vulnerable to acute-on-chronic kidney injury and severe systemic inflammation; however, these retrospective observational data cannot confirm a direct protective causal effect of haemodialysis. These findings have direct clinical implications: healthcare systems in Central Asia and comparable transitional settings should prioritise early nephrological consultation, aggressive volume and metabolic monitoring, and a low threshold for initiating renal replacement therapy in conservatively managed CKD patients hospitalised with COVID-19. Future research should validate these findings in multi-centre cohorts, examine the impact of vaccination and newer variants, and evaluate whether earlier initiation of dialysis in conservatively managed CKD patients with severe COVID-19 improves survival. Future studies should include sensitivity analyses under different analytical assumptions to further validate these findings.

## Figures and Tables

**Table 1:** Baseline demographic and clinical characteristics of hospitalised COVID-19 patients stratified by chronic kidney disease status (*n* = 891).

Characteristic	Total (*N* = 891)	No CKD (*n* = 598)	CKD no HD (*n* = 116)	CKD on HD (*n* = 177)	*p*-Value
Age, years	61 [50–71]	60 [50–71]	66 [57–73]	59 [48–66]	<0.001
Sex: female, *n*/*N* (%)	459/891 (51.5%)	330/598 (55.2%)	60/116 (51.7%)	69/177 (39%)	0.001
Sex: male, *n*/*N* (%)	432/891 (48.5%)	268/598 (44.8%)	56/116 (48.3%)	108/177 (61%)	—
Days of illness before admission	4 [2–7]	5 [2–7]	4 [2–7]	4 [2–7]	0.085
Hospital stay, days	8 [5–11]	7 [5–10]	8 [4–12]	10 [7–13]	<0.001
SpO_2_ on admission, %	90 [88–90]	90 [86–90]	90 [90–95]	90 [90–90]	0.005
Respiratory rate on admission	20 [19–24]	21 [19–24]	20 [19–24]	20 [19–22]	0.240
COVID-19 severity: mild, *n*/*N* (%)	1/884 (0.1%)	1/597 (0.2%)	0/113 (0%)	0/174 (0%)	—
COVID-19 severity: moderate, *n*/*N* (%)	124/884 (14%)	101/597 (16.9%)	10/113 (8.8%)	13/174 (7.5%)	—
COVID-19 severity: severe, *n*/*N* (%)	727/884 (82.2%)	481/597 (80.6%)	90/113 (79.6%)	156/174 (89.7%)	—
COVID-19 severity: extremely severe, *n*/*N* (%)	32/884 (3.6%)	14/597 (2.3%)	13/113 (11.5%)	5/174 (2.9%)	—
Obesity, *n*/*N* (%)	56/891 (6.3%)	35/595 (5.9%)	14/115 (12.2%)	7/177 (4%)	0.042
Diabetes mellitus, *n*/*N* (%)	299/891 (33.6%)	165/598 (27.6%)	70/115 (60.9%)	64/177 (36.2%)	<0.001
Arterial hypertension, *n*/*N* (%)	564/891 (63.3%)	301/598 (50.3%)	100/116 (86.2%)	163/177 (92.1%)	<0.001
Chronic heart failure, *n*/*N* (%)	378/891 (42.4%)	201/598 (33.6%)	78/116 (67.2%)	99/177 (55.9%)	<0.001
COPD, *n*/*N* (%)	24/891 (2.7%)	15/598 (2.5%)	3/116 (2.6%)	6/177 (3.4%)	0.777
History of stroke, *n*/*N* (%)	122/891 (13.7%)	77/597 (12.9%)	27/116 (23.3%)	18/177 (10.2%)	0.006
History of myocardial infarction, *n*/*N* (%)	34/891 (3.8%)	18/598 (3%)	10/115 (8.7%)	6/177 (3.4%)	0.022

Data are presented as median [lower limit (Q1)—upper limit (Q3) of the interquartile range] or *n*/*N* (%). COPD: chronic obstructive pulmonary disease; CKD: chronic kidney disease; HD: haemodialysis; SpO_2_: peripheral oxygen saturation. *p*-values from the Kruskal–Wallis test (continuous) or *χ*^2^/Fisher’s exact test (categorical). Significance criteria: *p* < 0.05, *p* < 0.01, and *p* < 0.001. Some column denominators vary due to missing values.

**Table 2:** Treatment, respiratory support, and intensive care based on CKD status.

Treatment	Total (*N* = 891)	No CKD (*n* = 598)	CKD no HD (*n* = 116)	CKD on HD (*n* = 177)	*p*-Value
Oxygen therapy	733/891 (82.2%)	466/598 (77.9%)	102/116 (87.9%)	165/177 (93.2%)	<0.001
Non-invasive ventilation (NIV)	73/891 (8.2%)	26/596 (4.4%)	16/113 (14.2%)	31/177 (17.5%)	<0.001
Invasive mechanical ventilation (IMV)	147/891 (16.5%)	74/597 (12.4%)	33/116 (28.4%)	40/176 (22.7%)	<0.001
ICU admission/transfer	188/703 (26.7%)	92/437 (21.1%)	43/104 (41.3%)	53/162 (32.7%)	<0.001
Vasopressor/adrenergic support	189/891 (21.1%)	100/598 (16.7%)	45/116 (38.8%)	44/177 (24.9%)	<0.001
Antiviral therapy	15/891 (1.7%)	14/598 (2.3%)	0/116 (0%)	1/177 (0.6%)	0.114
Favipiravir	1/891 (0.1%)	0/598 (0%)	0/116 (0%)	1/177 (0.6%)	0.328
Remdesivir	2/891 (0.2%)	2/598 (0.3%)	0/116 (0%)	0/177 (0%)	1.000
Antibacterial therapy	862/891 (96.4%)	573/598 (95.8%)	112/116 (96.6%)	177/177 (100%)	0.002
Heparin (unfractionated)	291/891 (32.6%)	208/598 (34.8%)	31/116 (26.7%)	52/177 (29.4%)	0.155
Enoxaparin	463/891 (51.8%)	272/598 (45.5%)	73/116 (62.9%)	118/177 (66.7%)	<0.001
Glucocorticoids	736/891 (82.4%)	486/598 (81.3%)	98/116 (84.5%)	152/177 (85.9%)	0.281

Data are presented as *n*/*N* (%). ICU: intensive care unit. *p*-values from *χ*^2^ or Fisher’s exact test. Significance criteria: *p* < 0.05, *p* < 0.01, and *p* < 0.001.

**Table 3:** Admission and peak laboratory values by CKD status.

Laboratory Parameter	Total	No CKD	CKD no HD	CKD on HD	*p*-Value
Platelets at admission, ×10^9^/L	204 [152–269.2]	213 [160–285]	208 [160.8–281.5]	175.5 [123–218]	<0.001
CRP at admission, mg/L	42.1 [9.4–165.6]	31.7 [8.1–138.5]	37.9 [7.5–171.7]	76.8 [18.4–244.6]	<0.001
CRP, peak, mg/L	69.6 [12.8–230.5]	44.4 [10–178.5]	110.4 [10.9–271.2]	168.3 [28.9–327.4]	<0.001
Ferritin, peak, µg/L	544 [246.8–1216.2]	383.8 [181.5–772.3]	635.9 [296.7–1213.9]	1109 [603.5–2158.4]	<0.001
Procalcitonin, peak, ng/mL	0.3 [0.1–1.5]	0.1 [0.1–0.3]	0.5 [0.2–1.5]	1.5 [0.6–2.9]	<0.001
D-dimer, peak, mg/L	1.8 [0.8–4.5]	1.3 [0.6–3.7]	2.8 [1.2–7.1]	2.4 [1.2–5.9]	<0.001
Fibrinogen, peak, g/L	4.9 [3.8–6.2]	4.8 [3.8–6.1]	5 [4.0–6.2]	5.1 [4.0–6.4]	0.284
Creatinine at admission, µmol/L	91 [67–186.8]	77 [60–94]	139 [101.5–214.5]	584 [372–788.2]	<0.001
Creatinine, peak, µmol/L	93 [68–213]	78 [61–97]	146 [107.5–222]	652.5 [480.8–836.2]	<0.001
Creatinine, last value, µmol/L	81 [61–160.8]	68 [56–84]	116 [85–187.5]	452.5 [298.5–587.2]	<0.001
Urea at admission, mmol/L	6.7 [4.3–12.7]	5.2 [3.7–7.1]	11.9 [7.8–17.4]	18.4 [11.2–25.1]	<0.001
Urea, peak, mmol/L	7.9 [4.9–16.4]	5.6 [4.1–8.2]	14.2 [10.5–20.9]	22.9 [18.1–31]	<0.001
Albumin at admission, g/L	31.6 [27.9–37.2]	31.1 [28.4–35.4]	29.6 [25.6–33.5]	33.1 [28.5–39]	0.009
ALT, peak, U/L	26.4 [15.4–46.4]	30.6 [16.6–53.8]	21.2 [13–35.3]	21.4 [13.8–35.4]	<0.001
AST, peak, U/L	65 [53–75.6]	65 [53–77]	68.5 [59.8–79.2]	62 [51–71]	0.01
LDH, peak, U/L	279 [209–393.5]	251.5 [201.2–354.2]	309.5 [225.8–495.5]	330 [242–427]	<0.001
Haemoglobin at admission, g/L	126 [107–140]	131 [116–143]	122.5 [97.8–141]	105 [91.5–119]	<0.001

Data are presented as median [IQR]. ALT: alanine aminotransferase; AST: aspartate aminotransferase; CRP: C-reactive protein; LDH: lactate dehydrogenase. *p*-values from the Kruskal–Wallis test.

**Table 4:** Hospital clinical outcomes of COVID-19 patients by CKD status.

Outcome	Total	No CKD	CKD no HD	CKD on HD	*p*-Value
Severe or extremely severe COVID-19	759/884 (85.9%)	495/597 (82.9%)	103/113 (91.2%)	161/174 (92.5%)	0.001
Acute kidney injury (AKI)	132/891 (14.8%)	69/587 (11.8%)	41/115 (35.7%)	22/176 (12.5%)	<0.001
Worsening renal function by creatinine	38/852 (4.5%)	12/561 (2.1%)	7/115 (6.1%)	19/176 (10.8%)	<0.001
Creatinine decrease after peak	428/852 (50.2%)	229/561 (40.8%)	67/115 (58.3%)	132/176 (75%)	<0.001
ARDS	118/891 (13.2%)	63/598 (10.5%)	26/116 (22.4%)	29/177 (16.4%)	0.001
Multi-organ dysfunction syndrome (MODS)	149/891 (16.7%)	72/598 (12%)	40/116 (34.5%)	37/176 (21%)	<0.001
ICU admission/treatment	188/703 (26.7%)	92/437 (21.1%)	43/104 (41.3%)	53/162 (32.7%)	<0.001
Non-invasive ventilation	73/891 (8.2%)	26/596 (4.4%)	16/113 (14.2%)	31/177 (17.5%)	<0.001
Invasive mechanical ventilation	147/891 (16.5%)	74/597 (12.4%)	33/116 (28.4%)	40/176 (22.7%)	<0.001
Vasopressor/adrenergic support	189/891 (21.1%)	100/598 (16.7%)	45/116 (38.8%)	44/177 (24.9%)	<0.001
In-hospital death	159/891 (17.8%)	81/597 (13.6%)	40/115 (34.8%)	38/175 (21.7%)	<0.001
Hospital length of stay, days	8 [5–11]	7 [5–10]	8 [4–12]	10 [7–13]	<0.001

Data are presented as *n*/*N* (%) or median [IQR]. ARDS: acute respiratory distress syndrome; MODS: multi-organ dysfunction syndrome; ICU: intensive care unit. *p*-values from the Kruskal–Wallis test (length of stay) or χ^2^/Fisher’s exact test (categorical).

**Table 5:** Multivariate logistic regression models of factors associated with in-hospital mortality.

Predictor	OR	95% CI	*p*-Value
Model 1: baseline clinical factors (*n* = 329)			
Age (per year)	1.03	1.01–1.06	0.016
Sex: male	0.89	0.48–1.65	0.718
CKD status: CKD no HD	3.23	1.38–7.63	0.007
CKD status: CKD on HD	1.03	0.44–2.31	0.951
Diabetes mellitus	1.43	0.75–2.68	0.27
Arterial hypertension	1.03	0.43–2.48	0.944
Chronic heart failure	1.54	0.70–3.44	0.288
Obesity	1.54	0.47–4.59	0.455
SpO_2_ on admission, %	1	0.99–1.02	0.578
Day of illness at admission	0.96	0.89–1.02	0.262
Model 2: extended clinical–laboratory model (*n* = 252)			
Age (per year)	0.82	0.47–1.01	0.206
Sex: male	0.08	0–7.56	0.343
CKD status: CKD no HD	7.2	0.01–59,269.51	0.541
Diabetes mellitus	4.06	0.03–587.02	0.502
Arterial hypertension	132.5	0.98–268,523,178.63	0.192
Chronic heart failure	0.7	0–564.06	0.912
SpO_2_ on admission, %	1	0.86–1.15	0.97
Acute kidney injury	0	0–0.33	0.159
Peak creatinine	0.96	0.86–1	0.193
Peak CRP	0.97	0.91–0.99	0.181
Peak D-dimer	1.12	0.97–1.51	0.163
Invasive mechanical ventilation	537.55	1.55–1,030,606,343.76	0.142
ARDS	1,412,853.2	530.36–5.06 × 10^17^	0.065
MODS	5.51 × 10^10^	3.80 × 10^4^–7.25 × 10^29^	0.093

OR: odds ratio; CI: confidence interval. Model 1: baseline clinical factors available at admission. Model 2: extended model additionally incorporating renal, inflammatory, and in-hospital severity markers. Wide CIs in Model 2 reflect quasi-complete separation due to high fatality of multi-organ failure; estimates should be interpreted qualitatively. Reference category for CKD status is “No CKD”.

**Table 6:** Multivariate logistic regression model of factors associated with acute kidney injury (AKI).

Predictor	OR	95% CI	*p*-Value
Age (per year)	0.99	0.96–1.02	0.509
Sex: male	0.91	0.45–1.84	0.792
CKD status: CKD no HD	4.12	1.73–10.03	0.002
CKD status: CKD on HD	0.71	0.25–1.87	0.493
Diabetes mellitus	1.72	0.85–3.53	0.132
Arterial hypertension	0.69	0.23–2.01	0.495
Chronic heart failure	2.43	0.94–6.74	0.074
SpO_2_ on admission, %	0.93	NA–1	0.611
Peak CRP	1	NA–1	0.759
Peak D-dimer	1.02	1–1.05	0.067
Glucocorticoids	0.85	0.24–3.61	0.81

Model *n* = 255. AUC = 0.759; McFadden pseudo-*R*^2^ = 0.155; maximum VIF = 1.40. OR: odds ratio; CI: confidence interval; NA: not applicable (boundary estimate due to zero-cell counts or quasi-complete separation). Reference category for CKD status is “No CKD”.

**Table 7:** Multivariate logistic regression model of factors associated with ICU admission/treatment.

Predictor	OR	95% CI	*p*-Value
Age (per year)	1.03	1–1.06	0.045
Sex: male	0.81	0.37–1.75	0.6
CKD status: CKD no HD	1.79	0.63–5.07	0.273
CKD status: CKD on HD	0.61	0.14–2.44	0.502
Diabetes mellitus	1.15	0.54–2.44	0.711
Arterial hypertension	0.99	0.34–2.95	0.979
SpO_2_ on admission, %	1	0.98–1.02	0.947
Acute kidney injury	4.55	1.95–11.04	<0.001
Peak CRP	1	NA–1	0.773
Peak D-dimer	1.04	1.01–1.09	0.034
Peak creatinine	1	1–1	0.11

Model *n* = 181. AUC = 0.823 (95% CI: 0.762–0.884); McFadden pseudo-*R*^2^ = 0.195; maximum VIF = 1.84. OR: odds ratio; CI: confidence interval; NA: not applicable (boundary estimate due to zero-cell counts or quasi-complete separation). Reference category for CKD status is “No CKD”.

**Table 8:** Diagnostics of the multivariate logistic regression models.

Model	*N*	AIC	McFadden Pseudo-*R*^2^	AUC	Max VIF
Mortality—baseline (Model 1)	329	303.4	0.16	0.755	1.34
Mortality—extended (Model 2)	252	50.53	0.393	0.999	10.79
Acute kidney injury	255	236.77	0.155	0.759	1.4
ICU admission	181	209.96	0.195	0.823	1.84

AIC: Akaike Information Criterion; AUC: area under the receiver operating characteristic curve; VIF: variance inflation factor. Higher AUC indicates better discrimination; lower AIC indicates better fit; VIF > 5 suggests multicollinearity. Caution: Metrics for the extended mortality Model 2 are driven by quasi-complete separation and severe multicollinearity. Model 2 is an exploratory analysis only and should not be interpreted as a validated predictive model. These statistics should not be interpreted as indicative of a valid or stable predictive model.

## Data Availability

The de-identified dataset analysed during the current study is available from the corresponding author upon reasonable request.

## Abbreviations

The following abbreviations are used in this manuscript:

AKI	Acute kidney injury
AIC	Akaike Information Criterion
AUC	Area under the receiver operating characteristic curve
ARDS	Acute respiratory distress syndrome
CI	Confidence interval
CKD	Chronic kidney disease
CKD-EPI	Chronic Kidney Disease Epidemiology Collaboration
COPD	Chronic obstructive pulmonary disease
CRP	C-reactive protein
eGFR	Estimated glomerular filtration rate
EMR	Electronic medical record
ESRD	End-stage renal disease
HD	Haemodialysis
ICU	Intensive care unit
IQR	Interquartile range
IMV	Invasive mechanical ventilation
KDIGO	Kidney Disease: Improving Global Outcomes
LDH	Lactate dehydrogenase
MODS	Multi-organ dysfunction syndrome
NIV	Non-invasive ventilation
OR	Odds ratio
RRT	Renal replacement therapy
SpO_2_	Peripheral oxygen saturation
VIF	Variance inflation factor
